# Comparing token-based and trajectory-based vowel space metrics for assessing speech in Parkinson’s disease

**DOI:** 10.1371/journal.pone.0353680

**Published:** 2026-07-21

**Authors:** Defne Abur, Megan Cushman, Courtney J. Dunsmuir, Cara E. Stepp

**Affiliations:** 1 Department of Speech, Language, and Hearing Sciences, Boston University, Boston, Massachusetts, United States of America; 2 Center for Language and Cognition Groningen, University of Groningen, Groningen, the Netherlands; 3 Research School for Behavioral and Cognitive Neurosciences Groningen, University of Groningen, Groningen, the Netherlands; 4 Department of Biomedical Engineering, Boston University, Boston, Massachusetts, United States of America; 5 Department of Otolaryngology – Head and Neck Surgery, Boston University School of Medicine, Boston, Massachusetts, United States of America; National Taiwan Normal University, TAIWAN

## Abstract

This study assessed the ability of three acoustic measures of vowel space, two token-based and one trajectory-based, to quantify speech differences in people with Parkinson’s disease compared to controls and correlations with speech intelligibility. Fifty-six speakers (28 people with Parkinson’s and 28 controls) read a custom reading passage containing corner vowels. For token-based measures, the triangular vowel space area (tVSA) in kHz^2^ and the unitless ratio of vowel articulation index (VAI) were calculated. For the trajectory-based measure, the articulatory-acoustic vowel space (AAVS) in kHz^2^ was calculated. Speakers also read five unique sentences varying in word length, which were used to collect speech intelligibility ratings. Analyses of Variance revealed that all vowel space measures yielded group differences. Binary logistic regression revealed that only the token-based measures were able to statistically discriminate PwPD from controls, however all models performed poorly (R^2^ < 0.113). Linear regressions showed that token-based metrics were statistically related to intelligibility and that the VAI yielded the best model for the current sample. The results support that token-based measures are well-suited for quantifying changes in acoustic vowel space and perceived speech intelligibility in people with Parkinson’s. Future work should examine the generalizability of these findings to other motor speech disorders as well as across different speech stimuli.

## Introduction

Parkinson’s disease (PD) is a neurodegenerative disorder that impacts several areas of the brain and body, including those involved in speech production [1]. People with PD (PwPD) frequently experience changes to respiratory features (e.g., decreased vital capacity), vocal features (e.g., reduced prosody and loudness), and articulatory features (e.g., imprecise articulation) of speech that are present throughout the disease progression and do not clearly improve with dopaminergic medication (see [2] for a review). Articulatory changes in PwPD related to vowels warrant particular attention, since vowel working space (i.e., the range in first and second vowel formant frequencies, *F*_1_ and *F*_2_, used to produce vowels) is directly linked to how well a listener can understand someone’s speech [e.g., 3,4]. Further, vowel working space in PwPD has been associated with the time spent living with PD [5]. These studies suggest that careful examination of vowel working space in PwPD can inform speech-based disease tracking and symptom management.

Acoustic measures of vowel working space have extensively been used to quantify articulatory speech changes in PwPD and different methodologies have been proposed [e.g., 6–9]. These methods include both token-based metrics (i.e., *F*_1_ and *F*_2_ from single vowels) and trajectory-based metrics (i.e., *F*_1_ and *F*_2_ trajectories across all vocalic portions of connected speech) for vowel space analysis. The benefit of using single vowel sounds is that this method yields more stable *F*_1_ and *F*_2_ trajectories for acoustic analysis, whereas extracting *F*_1_ and *F*_2_ from connected speech is more difficult since unvoiced sounds and pauses need to be removed prior to analysis. On the other hand, *F*_1_ and *F*_2_ trajectories extracted from vocalic sounds across multiple sentences better reflect daily communication compared to isolated vowels. Regardless of the specific methodology used, reductions in acoustic measures of articulatory working space (which minimize the contrast between different vowels) are associated with declines in perceived speech intelligibility and clarity [e.g., 3,10,11]. These findings suggests that both token-based and trajectory-based measures of vowels are promising tools for tracking speech function in PwPD.

The most common token-based metrics that have been used to characterize vowel working space in PwPD are the quadrilateral or triangular vowel space area (VSA and tVSA) and the vowel articulation index (VAI) [e.g., 4,6,9,12,13]. These metrics can be extracted from prolonged, isolated productions of corner vowels that form the vowel space (e.g.,/i/,/u/,/ɑ/, and/ae/ in American English) or by using corner vowels produced in running speech to increase ecological validity [4,12,14]. The VSA uses all four corner vowels, whereas the tVSA and VAI use three (/i/,/u/,/ɑ/ in American English). The VSA and tVSA are calculated as the area in *F*_1_ and *F*_2_ space across the respective corner vowels in kHz^2^. These metrics reflect the size of the articulatory working space, such that a larger VSA or tVSA value corresponds to a larger articulatory working space. The same *F*_1_ and *F*_2_ values of the three corner vowels are used to calculate the unitless ratio yielding VAI, in which a larger value also indicates a larger range in articulatory working space [9].

The VSA, tVSA, and VAI have all shown sensitivity to speech in PwPD compared to controls [e.g., 6,9,13,15–18]. Yet there are some studies of VSA in PwPD that have reported no statistical differences relative to controls [11,12,19]. The lack of group differences in these studies may be related to the smaller samples assessed (10 speakers with PD [11]), a milder presentation of speech symptoms in the participants with PD [12], or the lack of sensitivity of the VSA metric to speech changes in PwPD. In support of this last possibility, there is evidence of differential sensitivity to speech changes in PwPD among token-based measures of vowel space. Studies investigating both tVSA and VAI in PwPD have found that VAI was sensitive to group differences whereas tVSA was not [9,15]. This could be a result of VAI accounting for differences in baseline formant values across speakers since it is a unitless ratio derived from formant values. Instead, the tVSA and VSA are calculated using the speaker’s formant values in Hz, which can influence comparisons across groups with different ranges in baseline formant values (e.g., average values for males compared to females). These results highlight the need to assess speech in PwPD who have a wide range in speech severity and to consider factors that may affect the speaker’s baseline formant values (e.g., the speaker’s sex) in investigations using token-based vowel space metrics.

In addition to token-based metrics, some studies have proposed trajectory-based metrics of vowel space to quantify speech differences in PwPD compared to controls to assess formant productions that encompass more than just corner vowels. These include the Vowel Space Density (VSD; [20]) and the Articulatory-acoustic Vowel Space (AAVS; [7]), which use formant trajectories in Hz to provide information about formant space density and variance, respectively. While the VSD can identify denser regions of the formant space that speakers are using most often [14,20], the AAVS is a measure of variance in the formant space that yields a ‘global articulatory–acoustic range of motion or working space used for an entire utterance’ [7]. Given that both VSD and AAVS metrics use a speaker’s raw formant values in Hz, it is important to consider sex as a possible factor in their analysis [14].

When factoring for sex, the VSD and AAVS have both shown sensitivity to speech in PwPD compared to controls [7,21,22]. Although one study found the VSD was more sensitive to speech changes in PwPD than the AAVS during habitual reading [14], other preliminary work found that the AAVS was sensitive to speech changes in PwPD for both habitual reading and semi-spontaneous speech tasks (“The Cookie Theft Picture” description task [21]). Additionally, the AAVS has shown sensitivity to speech differences in PwPD across languages (i.e., English compared to preliminary work in Dutch [7,21,22]) and has been associated with perceptual ratings of speech clarity [7]. There is also work showing that AAVS did not differentiate between a group of PwPD and controls [23]; however, the PwPD included in the study were rated as having mild dysarthria, which could have driven the lack of group differences. Together, these studies demonstrate that AAVS shows promise as a tool to quantify speech changes in PwPD and that speech symptom severity is an important consideration in investigations.

Regardless of whether vowel space metrics are trajectory-based or token-based, the stimuli used to elicit the vowel productions remain an important consideration. For token-based vowel metrics, the speech stimuli must contain instances of corner vowels as mentioned earlier. These instances of corner vowels have been calculated across single, prolonged vowels in PwPD [e.g., 13,24], which yield clear formant traces for applications such as automated speech assessments [25]. However, token-based measures of vowel space appear to be most sensitive to group differences in PwPD and controls when extracted from sentences compared to isolated vowels [17]. Thus, other studies have assessed corner vowels in PwPD extracted from connected speech stimuli [e.g., 12,14,15]. Although connected speech can increase susceptibility of vowels to co-articulation effects, it also increases ecological validity for assessing daily communication.

For trajectory-based metrics of vowel space, speech stimuli are always from connected speech and can include a wide variety of vowels for a comprehensive examination of the range for each type of vowel. Although this results in a more complete representation of a speaker’s vowel productions, the acoustic values will be affected by the specific sentences themselves (depending on which vowels are included and what the articulatory transitions are). For this reason, trajectory-based metrics of vowel space in PwPD are not directly comparable across prior works that use different stimuli or languages [e.g., 4, 14, 21, 22]. The use of a standardized speech stimulus for trajectory-based vowel space measures, e.g., a reading passage with a specific set of vowel transitions for a given language, is necessary for studies to be more comparable within language groups.

Although various token-based and trajectory-based metrics have been employed to quantify acoustic changes in vowel space area in PwPD compared to controls, it is not clear which of these acoustic measures is optimal for identifying articulatory differences and tracking perceived speech intelligibility in PwPD. Toward increasing consistency in research and optimizing clinical speech assessments for PwPD, the current work aimed to how different acoustic metrics of vowel space perform when discriminating Parkinsonian speech and predicting intelligibility. Given prior findings that vowel space measures may not be sensitive in samples with mild speech symptoms [23] or with small sample sizes [14], the current work also aimed to assess a larger group of speakers (N = 28 per group) across a wide range in speech symptoms (intelligibility ratings from mild to severe). Two token-based metrics (tVSA and VAI) and one trajectory-based metric (AAVS) were included based on their demonstrated effectiveness in distinguishing speech changes due to PD across a variety of stimuli [e.g., VAI: 5,7, tVSA: 16, and AAVS: 22]. Since both traditional VSA and tVSA have shown sensitivity to speech differences in PwPD [13,16], the tVSA was preferred in the current study as it requires less vowel stimuli (three compared to four vowels). Given that VAI has also shown greater sensitivity to speech changes in PwPD compared to tVSA [9,15], both of these token-based measures were included in the study. For the trajectory-based measure, the AAVS was selected given that it has shown sensitivity to speech differences in PwPD across different stimuli, speaking styles, and languages [7,21,22].

Thus, the goal of the current work was to assess the ability of three established acoustic measures for tracking changes in vowel space in PwPD (the tVSA, VAI, and AAVS), spanning token-based and trajectory-based methods and extracted from the same type of speech stimuli, to quantify speech differences between PwPD and control speakers and reflect perceptual speech intelligibility. The first aim of this study was to determine whether each of the three acoustic measures of vowel space yielded group-level differences between PwPD and control speakers. The second aim was to determine how the metrics performed in determining group status: PwPD or control. The third aim was to determine whether the acoustic metrics were associated with perceptual speech intelligibility.

## Materials and methods

### Participants

All study participants completed written informed consent in compliance with the Boston University Institutional Review Board. The study included 56 speakers and five listeners. The data were collected during a larger study with the same two participant groups [26]. The speakers were recruited between January 27^th^ 2017 – November 20^th^ 2019 and the listeners were recruited between February 10^th^ – February 27^th^ 2020.

A total of 56 speakers participated in the study across two groups. The first group included 28 cisgender persons with Parkinson’s disease (PwPD; 11 females, 17 males) aged 45–73 years (M = 61.9 years, SD = 7.8 years). The second group of participants included 28 control speakers (who were sex-, gender- and hearing-matched to the PwPD group) aged 48–81 years (M = 63.9 years, SD = 8.5 years). All speakers reported American English as their native language. All PwPD were diagnosed with idiopathic Parkinson’s disease (PD) by a neurologist (ranging 1–21 years post-diagnosis) and were receiving dopaminergic medication at the time of data collection. The PwPD included in the study were not asked to go off their medication cycle to maintain ecological validity and examine speech symptoms as they would present in daily life. No participants were enrolled in speech therapy at the time of the study. PwPD also completed the Movement Disorder Society-Sponsored Revision of Unified Parkinson’s disease Rating Scale Part III (Motor symptom) assessment and had scores ranging from 17–64 (< 33 indicating mild motor impairment and > 59 indicating severe motor impairment). Control participants denied any neurological, speech, hearing, cognitive, or language disorders. All speakers underwent an audiometric hearing screening (Grason-Stadler GSI 18, Grason-Stadler, Eden Prairie, MN). All but four speakers passed a hearing threshold test within the normal range for older adults at all frequencies (using a 25 dB HL cutoff at 1000 Hz and below and a 40 dB HL cutoff at 2000 Hz and 4000 Hz [27]). The remaining four participants (two in each speaker group) did not pass the threshold test at one frequency and were hearing-matched across groups.

Five cisgender listeners aged 18–29 years (4 female, 1 male), who reported no experience rating disordered speech, completed ratings of speech intelligibility. The number of listeners were chosen given that prior work found five unfamiliar listeners showed acceptable intelligibility ratings for speakers with Parkinson’s [28]. Namely, five listeners using a visual analog scale (VAS) task to rate 11 sentences per speaker showed reliable intelligibility estimates (i.e., with a 2% margin of error, which is below clinical intelligibility changes of 7.06% reported pre- vs. post-therapy [29]). Other work has reported that five sentences per speaker, rated by at least three unfamiliar listeners, are enough to yield reliable intelligibility estimates in Parkinson’s [30]. Specifically, with at least five sentences per speaker and at least three unfamiliar listeners using a VAS task, intelligibility estimates remained accurate (i.e., average estimates that were below the minimally detectable change, or the smallest magnitude of change required to be considered real rather than measurement error [30]). Based on findings from these prior studies, five listeners rating five sentences using a VAS task were deemed appropriate for the current task. All listeners reported American English as their native language. All listeners passed a standard hearing screening (using a 25 dB HL cutoff at all tested frequencies [29]) and denied any history of speech, language, hearing, or neurological disorders.

### Data collection

**Fig 1:**
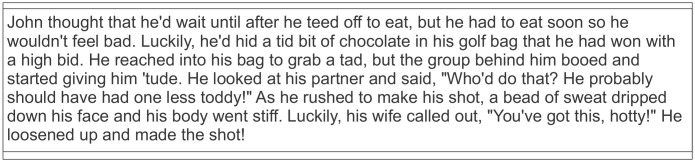
Corner vowels reading passage. A reading passage containing multiple instances of all American English corner vowels. For the vowel/ɑ/, the words ‘J**o**hn’, ‘pr**o**bably’, ‘t**o**ddy’, ‘sh**o**t’, ‘b**o**dy’, and ‘g**o**t’ were used. For the vowel/i/, the words ‘H**e’**d’, t**ee**d’, ‘**ea**t’, ‘f**ee**l’, ‘r**ea**ched’, and ‘b**ea**d’ were used. For the vowel/u/, the words ‘s**oo**n’, ‘gr**ou**p’, ‘b**oo**ed’, ‘’t**u**de’, wh**o**’, and ‘d**o**’ were used.

Speech was recorded using an omnidirectional microphone (MX153, Shure, Niles, IL) positioned 7 cm from the corner of the mouth at a 45° angle from the midline. The microphone signal was amplified via a Quadmic II pre-amplifier (RME Audio; Haimhausen, Germany) and digitized via an Ultralite-mk3 Hybrid soundcard (MOTU, Cambridge, MA). The acoustic recordings were collected using SONAR Artist (Cakewalk, Inc.) software. All speakers read a custom passage that contained multiple instances of all American English corner vowels (Fig 1). A connected speech sample was selected for this study to maintain ecological validity in the characterization of vowel space area. The same speech sample was used to calculate all measures of vowel space area to prevent confounds due to differences in token-based and trajectory-based stimuli, as done in prior work [4,14].

All but two speakers also read a randomly generated set of the Sentence Intelligibility Test [SIT; 31] for intelligibility ratings. Each SIT consisted of 11 sentences increasing in word count from five to 15 words (for example stimuli, see [32]). For intelligibility ratings, a representative subset was selected by choosing five even numbered sentences (Sentences 2, 4, 6, 8, and 10) so that they maintained the increasing word count (6 words to 14 words). For the two speakers who did not have recordings of the SIT, a separate set of five sentences was extracted from other speech tasks in the study ranging from 6–14 words to match the word count of the even numbered SIT sentences. The task lasted approximately 1.5 hours. Separate stimuli were used for the intelligibility ratings to prevent familiarization effects (i.e., so that listeners would not become biased in their ratings by hearing the same passage repeatedly). In addition, prior work found acoustic measures of vowel space were correlated with speech intelligibility on the same stimuli [4], thus the current work sought to extend this to differing stimuli. The current study assessed if the relationship between vowel space measures and intelligibility was maintained when calculated over differential stimuli from the same speaker.

For intelligibility ratings, the SIT recordings from the speaker groups were amplitude-normalized, mixed with multi-speaker babble from four male and four female speakers (who were not part of either speaker group), and presented to the listeners. The multi-speaker babble was mixed in with speech stimuli at a 1-dB signal-to-noise ratio (based on pilot testing at 0, 1, 1.5, and 2 dB) to prevent ceiling effects and increase ecological validity [33,34]. The pilot testing consisted of three listeners rating all stimuli at the different signal-to-noise ratios and assessing the first level without noticeable ceiling effects (i.e., majority of stimuli being 100% intelligible). All stimuli were presented to listeners in a randomized order via headphones (Sennheiser 280 Pro HD). The headphone output was set at 60–70 dB SPL as in prior work [28] based on the typical intensity range of conversational speech [35]. These intelligibility ratings were previously published as part of a larger study [26].

During the rating task, listeners were provided with a definition of speech intelligibility as “the degree to which a speaker’s message can be recovered by a listener” and asked to use a visual analog scale (VAS) for their ratings [36]. The VAS for speech intelligibility ranged from 0 (minimum) to 100 (maximum). Based on prior work which found that both orthographic transcription and visual analog scale measures of speech intelligibility demonstrated relations to vowel space metrics [4], the current work chose to use visual analog scale ratings to reduce the rating time burden on listeners [28]. The definition for intelligibility remained on a computer screen next to the participant for the duration of the study. Each listener was presented with five sentences from each speaker in the PwPD and control groups followed by a randomly selected 10% of repeated sentences for intra-rater reliability. This resulted in 308 sentence ratings per listener. Intra-rater and interrater reliabilities were calculated via two-way mixed effects intraclass correlations (ICCs) across listeners.

### Data analysis

All measures of vowel space were extracted from the same speech stimuli using the custom reading passage containing all American English corner vowels (Fig 1). The tVSA and VAI were extracted from vowels at the word-level, whereas the AAVS was calculated for the first two sentences of the passage. Thus, the tVSA and VAI were calculated from each instance of the three corner vowels in American English (/ɑ/,/i/, and/u/) and the AAVS was calculated from the full *F*_1_ and *F*_2_ trajectories in the first two sentences of the passage.

For the token-based measures (tVSA and VAI), six instances of each vowel (/ɑ/,/i/, and/u/) were extracted from specific words containing the vocalic nuclei using Praat software [37]. For the vowel/ɑ/, the words ‘J**o**hn’, ‘pr**o**bably’, ‘t**o**ddy’, ‘sh**o**t’, ‘b**o**dy’, and ‘g**o**t’ were used (six tokens). For the vowel/i/, the words ‘H**e’**d’, t**ee**d’, ‘**ea**t’, ‘f**ee**l’, ‘r**ea**ched’, and ‘b**ea**d’ were used (six tokens). For the vowel/u/, the words ‘s**oo**n’, ‘gr**ou**p’, ‘b**oo**ed’, ‘’t**u**de’, wh**o**’, and ‘d**o**’ were used (six tokens). Since the vowels were embedded in words during connected speech, the maximum stable region of the vowel was used for analysis. The maximum stable portion was determined visually and evaluated by three researchers and the findings were compared. For any discrepancies, formant tracking settings were discussed as a team to finalize the parameters. For each vowel instance, the average first formant (*F*_*1*_) and second formant (*F*_*2*_) values were calculated in hertz (Hz) across the selected region by using the ‘Get Formant’ feature in Praat. Formant estimation was optimized for each speaker using the formant setting in Praat (i.e., number of formants, maximum formant value, and window length). Individualized settings were saved for each speaker and reviewed by all three analyzers to ensure agreement. The *F*_*1*_ and *F*_*2*_ values across all instances were averaged by vowel, yielding one *F*_*1*_ and one *F*_*2*_ value in for each vowel that was used to calculate the tVSA (abs(*F*_1_/i/ * (*F*_2_/ɑ/ − *F*_2_/u/) + *F*_1_/ɑ/ * (*F*_2_/u/ − *F*_2_/i/) + *F*_1_/u/ * (*F*_2_/i/ − *F*_2_/ɑ/))/2 based on [9]) with units of kHz^2^. The tVSA reflects the size of the articulatory working space, thus, a larger value corresponds to a larger articulatory working space. The same *F*_1_ and *F*_2_ values were used to calculate the unitless ratio yielding VAI ((*F*_2_/i/ + *F*_1_/ɑ/)/ (*F*_1_/i/ + *F*_2_/ɑ/ + *F*_1_/u/ + *F*_2_/u/) based on [9]). For VAI, a larger value also indicates a larger range in articulatory working space with typical speech expected to have a value around 1.

For the trajectory-based measure (AAVS), the first two sentences of the custom reading passage were used (Fig 1). All non-vocalic portions of the speech stimuli were removed using a custom MATLAB [38] script interfacing with a Praat script provided in in prior work [39]. The output files were manually checked to ensure only vocalic portions were included. Using Praat, the F1 and F2 trajectories in Hz were then extracted from the samples with only vocalic portions. For each speaker, the formant settings from the token-based analysis were used as a starting point. If necessary, the formant settings were further adjusted and saved for each speaker. The final formant trajectories were reviewed by three analyzers to ensure agreement. Next, MATLAB was used to calculate the AAVS on the extracted formant trajectories using methods detailed in prior work [7,40]. The *F*_*1*_ and *F*_*2*_ trajectories in Hz that were extracted from Praat were read into MATLAB to calculate the square root of the generalized variance of the two formant trajectories as the AAVS in kHz^2^ for each speaker [7,14]. Since AAVS reflects the variance in *F*_*1*_ and *F*_*2*_ trajectories, a larger AAVs value is associated with a larger articulatory working space.

Speech intelligibility ratings were averaged across the five sentences for each speaker and then averaged across the set of listeners. Thus, one speech intelligibility rating was calculated for each speaker. Based on prior work, an intra-rater reliability of 0.6 was set as a cut-off value to include a listener’s ratings [41] and all listeners in the study met this criterion. The average intra-rater reliability ICC(3,1) was 0.851 and the interrater reliability ICC(3,1) was 0.769.

### Statistical analysis

Given that all metrics were extracted from the same stimuli, correlations were first assessed across the three vowel metrics. Pairwise Pearson Correlations showed that VAI and tVSA had a correlation of 0.772, AAVS and tVSA had a correlation of 0.771, and AAVS and VAI had a correlation of 0.496. To account for the correlation across the metrics, separate statistical models were built for each metric.

To address Aim 1, to determine how acoustic measures of vowel space area perform when quantifying differences between PwPD and controls, three analyses of variance (ANOVAs) were used to assess the effect of group (PwPD and controls), sex (female and male), and their interaction for tVSA, VAI, and AAVS. Since a main effect of sex has been reported in prior work examining vowel space area measures [7], sex was included as a variable in all ANOVAs. Factor effect sizes were quantified using the squared partial curvilinear correlation η_p_^2^. *Post-hoc* Tukey tests were used to determine the direction of relationships.

For Aim 2, to determine which acoustic measure of vowel space was best at discriminating between groups, three binary logistic regressions and resulting receiver operating characteristic (ROC) curves were used. Each model assessed one of the vowel space metrics (tVSA, VAI, and AAVS) as predictors of group (PwPD and controls). The area under the curve and *R*^2^ values from the binary logistic regressions were used to assess the models and determine how well each acoustic metric alone could predict group status.

For Aim 3, to determine which acoustic measure of vowel space was most related to speech intelligibility, a base model (with only group and sex as predictors) was compared to three linear regressions with the addition of each vowel metric (tVSA, VAI, and AAVS) as predictors of speech intelligibility ratings. Group (PwPD and controls) and sex (female and male) were used as categorical predictors in all models. All statistical analyses were performed using Minitab 19 software. For statistical analyses, a Bonferroni correction was applied to the alpha level of 0.05 to account for multiple comparisons; thus, the alpha level was set to 0.016.

## Results

**Fig 2:**
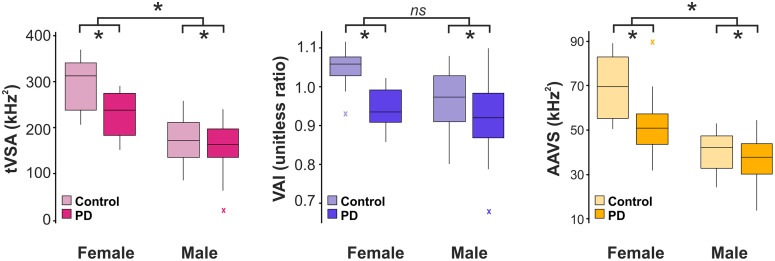
Vowel space metrics by speaker group. Boxplots of the triangular vowel space area (tVSA; left panel), vowel articulation index (VAI; middle panel), and the articulatory-acoustic vowel space (AAVS; right panel) are displayed by speaker group (Parkinson’s disease and controls) and by sex (female and male). PD = Parkinson’s disease. X = statistical outlier. * = statistically significant difference; ns = no statistically significant difference.

For the first study aim, ANOVAs were used to test the effect of group, sex, and their interaction on tVSA, VAI, and AAVS. The alpha level was set to 0.016 to correct for multiple comparisons. The ANOVAs all revealed a significant and large effect of group (PwPD and controls) on vowel space measures (Fig 2). Group was statistically significant in all models (VAI: *df* = 1, F = 11.50, *p* = 0.001, η_p_^2^ = 0.18, large effect size; tVSA: *df* = 1, F = 9.34, *p* = 0.004, η_p_^2^ = 0.15, large effect size; AAVS: *df* = 1, F = 9.31, *p* = 0.004, η_p_^2^ = 0.15, large effect size). *Post-hoc* Tukey tests revealed that PwPD had reduced values compared to controls across the three measures: VAI (PwPD: M = 0.94 and SD = 0.08, controls: M = 0.97, SD = 0.08), tVSA (PwPD: M = 193.28 kHz^2^, SD = 62.6 kHz^2^, controls: M = 236.15 kHz^2^, SD = 72.1 kHz^2^), and AAVS (PwPD: M = 4601.7 kHz^2^, SD = 412.0 kHz^2^, controls: M = 4803.7 kHz^2^, SD = 384.2 kHz^2^).

**Fig 3:**
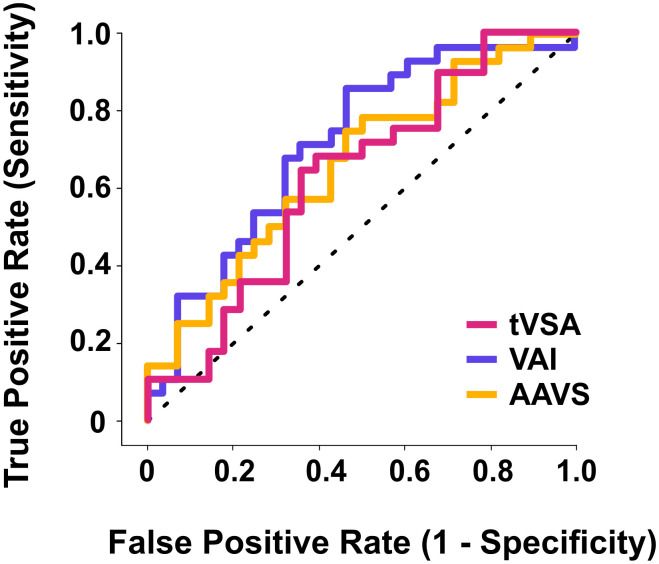
Receiver operating characteristic curves for vowel space metrics. Receiver operating characteristic curves for triangular vowel space area (tVSA; shown in pink), vowel articulation index (VAI; shown in purple), and the articulatory-acoustic vowel space (AAVS; shown in yellow) in distinguishing speaker group (Parkinson’s disease and controls).

The ANOVAs revealed a significant effect of sex on tVSA and AAVS values, with a large effect size (tVSA; *df* = 1, F = 47.95, *p* < 0.001, η_p_^2^ = 0.48, large effect size; AAVS: *df* = 1, F = 47.33, *p* < 0.001, η_p_^2^ = 0.48, large effect size). There was no significant effect of sex for the VAI (*p* = 0.02). *Post-hoc* Tukey tests revealed that females had larger values of vowel space metrics compared to males for both tVSA (females: M = 263.3 kHz^2^, SD = 60.5 kHz^2^; males: M = 166.15 kHz^2^, SD = 51.6 kHz^2^) and AAVS (females: M = 61.13 kHz^2^, SD = 16.54 kHz^2^; males: M = 38.82 kHz^2^, SD = 9.79 kHz^2^). There was no significant interaction between sex and group for AAVS (*p* = 0.042), tVSA (*p* = 0.08), or VAI (*p* = 0.15).,

For the second study aim, binary logistic regressions were used to yield the ROC curves and determine how well tVSA, VAI, and AAVS measures alone could predict group status (PwPD or control). All acoustic metrics were statistical predictors of group status. The binary logistic regressions (Fig 3) revealed the largest area under the curve for VAI (0.73), followed by tVSA (0.64) and then AAVS (0.63). The models were each validated using the Pearson goodness-of-fit tests to examine whether experimental findings matched the theoretical distribution of the data. For all three measures, the model passed the goodness-of-fit test. However, the binary logistic regression results suggested that all models had poor fits (*R*^2^ < 0.113).

**Fig 4:**
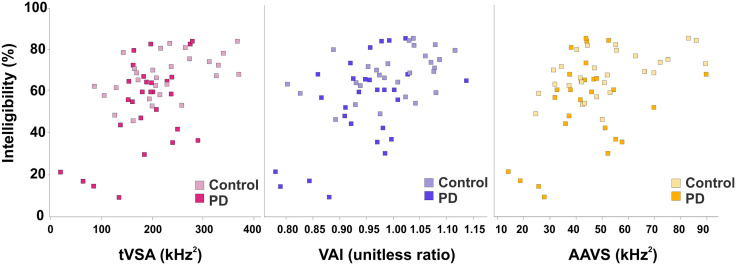
Relation between vowel metrics and speech intelligibility ratings. Scatterplot of perceptual speech intelligibility ratings for each speaker versus the triangular vowel space area (tVSA; left panel), vowel articulation index (VAI; middle panel), and the articulatory-acoustic vowel space (AAVS; right panel) values by group (Parkinson’s disease and controls). PD = Parkinson’s disease.

For the third aim of the study, linear regressions were used to assess whether tVSA, VAI, and AAVS were statistical predictors of speech intelligibility with group and sex as categorical predictors. Intelligibility ratings (Fig 4) ranged from 46% – 84% for the control group and from 9% – 84% in the PwPD group (S1 File). First a base model was build using only group and sex as predictors. Group (df = 1, F = 10.47, *p* = 0.002, VIF = 1.00) was a statistical predictor of intelligibility ratings and sex did not show a significant effect (*p* = 0.04, VIF = 1.00). The base model had an AICc of 476.7. Next, the base model was compared to three models where each vowel metric was added. For all models, the VIF values remained below 5. The VAI model showed that VAI was statistically associated with speech intelligibility (VAI: *df* = 1, F = 10.66, *p* = 0.002, VIF = 1.29), whereas group (*p* = 0.057, VIF = 1.18) and sex (*p* = 0.276, VIF = 1.11) were not significant predictors. The VAI model had an AICc of 468.7. The tVSA model showed that tVSA was also statistically associated with speech intelligibility (*df* = 1, F = 10.18, *p* = 0.002, VIF = 2.01) and group (*p* = 0.034, VIF = 1.14) and sex (*p* = 0.578, VIF = 1.18) were not significant predictors. The tVSA model had an AICc of 469.2. For the trajectory-based measure AAVS model, AAVS was not a statistical predictor of intelligibility (*p* = 0.022, VIF = 1.97) and neither were group (*p* = 0.021, VIF = 1.13) and sex (*p* = 0.976, VIF = 1.84). The AAVS model had an AICc of 473.4. In the VAI model, 35% of the variance in speech intelligibility ratings was explained by VAI values (*R* ^2^ = 0.349). In the tVSA model, 34.5% of the variance in intelligibility ratings was explained by tVSA values (*R* ^2^ = 0.345).

## Discussion

The current study assessed three acoustic measures of vowel space, calculated from the same connected speech stimuli, in their ability to (1) quantify speech differences in PwPD compared to controls, (2) discriminate speaker group, and (3) predict perceived speech intelligibility. The study included two token-based measures (the triangular vowel space area, tVSA; the vowel articulation index, VAI) and one trajectory-based measure (the articulatory-acoustic vowel space, AAVS). All metrics quantified speaker group differences and token-based metrics were statistically associated with speech intelligibility.

Given that speech changes in PwPD are very heterogenous across individuals and disease progression, the range in speech severity for the current study is important to consider alongside the results. Specifically, vowel space metrics may be less sensitive to milder speech symptoms in PwPD that do not differ substantially from age-matched controls [12]. For this reason, it is possible that the speech severity characteristics of the PwPD groups assessed across the literature can impact investigations, and comparisons, of vowel space area findings. The PwPD group assessed in this work had a relatively wide range in speech symptom severity as quantified by their intelligibility-in-noise ratings (mean = 54.6%, ranging from 9% to 84%) compared to the control group (mean = 68.1%, range from 46% to 84%). However, despite the large range in intelligibility, only four speakers appeared to have severe symptoms (i.e., intelligibility below 20%; Fig 4). Further, the intelligibility ratings for both the PwPD and control groups were somewhat lower than reports in prior work [42], which may result from different speech severity levels in both the PwPD and the control groups (e.g., presbyphonia) or different ratios of the multispeaker babble across studies. One prior VAS study of intelligibility in PwPD used the same multispeaker babble with a lower signal-to-noise of 0 (making the rating task harder) and still reported higher intelligibility ratings than in the current work [28], suggesting that signal-to-noise ratio did not drive the lower VAS ratings in the current work.

### Comparing token- and trajectory-based vowel space measures in PwPD and controls

For the first aim, all metrics yielded large effect sizes for group when quantifying differences between PwPD and controls. This result aligns with prior studies on token-based and trajectory-based measures of vowel space that have been able to quantify speech differences in PwPD [e.g., 6, 9, 13, 15, 16]..

Compared to prior work assessing VAI in PwPD, the values (across both male and female PwPD) in the current work (M = 0.93, SD = 0.08) align with earlier VAI findings in PwPD that were calculated using sentence readings in American English (M = 0.95 [13]; M = 0.96, SD = 0.08 [9]). However, when comparing the current VAI values to previous work in PwPD extracted from different types of stimuli and languages, the findings do not always align. Speech stimuli appear to have differential effects on group differences in VAI for PwPD. This is supported by an investigation of Czech speakers with PD and controls, which found that the VAI was more sensitive to group differences when extracted from sentences and monologue speech compared to isolated vowels [17]. In addition, VAI values in Belgian French speakers with PD extracted from phonations of isolated vowels were reported in a similar range to a study in Czech speakers with PD [16]. The PwPD in both studies had average VAI values from isolated vowels of approximately 1.1, which is higher than values found for the current control group of American English speakers and other reports of VAI extracted from sentences [9,15,17]. However, the study in Belgian French speakers with PD reported less than a 0.01-point difference between PwPD and controls [16] compared to a 0.1-point group difference in the group of Czech PwPD compared to controls [17]. Taken together, these findings suggest that there could be stimuli- and language-related factors, in addition to variability across speech impairments in PwPD, that influence VAI assessments.

When comparing the VAI values in the current work to the literature based, the influence of language is an important consideration. VAI to quantify speech changes in PwPD, studies that have used the same type of stimuli as the current work (sentences), but in different languages, show substantially different VAI findings. For example, prior studies of VAI extracted from sentence stimuli in German speakers with PD have yielded lower VAI measures compared to American English, regardless of group (M = 0.88 for controls and M = 0.82 for PwPD [15]; M = 0.77 for male controls, M = 0.88 for female controls, M = 0.75 for male PwPD, and M = 0.84 for female PwPD [5]). The same studies also yielded a smaller average VAI difference between speaker groups (0.02 to 0.06-point differences [15]) compared to the current work as well as prior findings for sentence VAI in PwPD in American English and Czech (~1.0 point difference [9,17]). A study in Finnish speakers with PD and controls reported similarly low VAI values, extracted from sentences in a passage reading, with minimal group differences (M = 0.76 for controls and M = 0.75 for PwPD [43]). Another study of VAI in Columbian Spanish speakers with PD, calculated from sentences, found even lower average values (M = 0.67 for PwPD with intelligibility changes and M = 0.75 for PwPD with typical intelligibility) compared to prior work [44]. These collective studies highlight the need for more multilingual and multi-stimuli investigations of vowel space measures in PwPD to determine measures that can best quantify speech changes for different languages.

When comparing the tVSA values found in the current work to prior work, they align well with a study assessing vowels from sentences in American English in controls and PwPD [9]. The previous study used the term “VSA” to refer to the same calculation as the current tVSA and found similar values for their older adult control group (M = 279.5 kHz^2^, SD = 68.8 kHz^2^) as reported here (M = 223.1 kHz^2^, SD = 78.1 kHz^2^). Although the PwPD group had higher tVSA values (M = 232.1 kHz^2^, SD = 96.1 kHz^2^ [9]) compared to this work (M = 185.5 kHz^2^, SD = 62.6 kHz^2^), the ranges are overlapping and higher average values could stem from milder speech severity in the previous study’s PwPD. Interestingly, both this work and the prior study in older adults and PwPD [9] reported larger values than previous reports of tVSA in young adult speakers during comfortable speech (M = 114.1 kHz^2^, SD = 51.8 kHz^2^ [14]). Although all three studies assessed tVSA extracted from sentence stimuli in American English, the specific phonetic contexts of the sentences may have influenced these set of findings. This is also supported by differences in the corresponding VAI values. In the young adult study [45], speakers had an average VAI of 0.83 during comfortable speech, whereas the current work found larger average VAI in PwPD (0.92), which is comparable to other average VAI reports in PwPD (0.95 [13], 0.96 [9]). Thus, this adds further evidence that the phonetic contexts of the stimuli may impact vowel space area comparisons within and across speaker groups.

Finally, the AAVS values in the current work are also comparable to prior reported ranges for PwPD and controls in kHz^2^ [7,14,23], despite the fact that some of the prior studies had fewer participants with PD (12 PwPD [7]; 15 PwPD [14]). More specifically, the current work’s AAVS findings in PwPD (M = 43.37 kHz^2^, SD = 14.9 kHz^2^) are comparable to prior AAVS findings in PwPD extracted from habitual sentence reading in American English (M = 39.9 kHz^2^, SD = 12.5 kHz^2^ [14]; M = 26.9–49.3 kHz^2^, SD = 5.7–1.6 kHz^2 [^7^]^; M = 38.9–53.7 kHz^2^, SD = 10.1–11.1 kHz^2^ [23]). Similarly, AAVS values for the group of older adult controls assessed here (M = 51.8 kHz^2^, SD = 17.8 kHz^2^) are within the range of prior findings (M = 46.7 kHz^2^, SD = 8.9 kHz^2^ [14]; M = 38.5–64.6 kHz^2^, SD = 5.2–9.8 kHz^2 [^7^]^; M = 39.7–59.1 kHz^2^, SD = 9.0–12.4 kHz^2^ [23]). This supports that AAVS is relatively consistent for cross-group comparisons, even with smaller sample sizes.

To compare the findings to investigations of AAVS in PwPD in mels^2^ [21,22], the AAVS for the current study was also calculated in mels^2^ by converting the *F*_1_ and *F*_2_ formant trajectories using the formula mels = 2595 * log10(1 + {formant value in Hz}/700) from MelFilter in [37]. The current findings for AAVS in mels^2^ (see S1 File) across the full group of PwPD (M = 19.2 mels^2^, SD = 5.3 mels^2^) were very similar to a previous study in PwPD from sentence readings in Dutch (M = 19.3 mels^2^, SD = 7.0 mels^2^ [22]). The findings are also relatively aligned with another sentence readings study in Dutch speakers that reported AAVS values in PwPD and controls by sex [21]. In PwPD, the current results for females (M = 20.9 mels^2^, SD = 5.8 mels^2^) and males (M = 18.0 mels^2^, SD = 4.7 mels^2^) are more strongly aligned with the previous study for males than females (M = 28.8 mels^2^, SD = 7.5 mels^2^ for female PwPD; M = 17.5 mels^2^, SD = 4.1 mels^2^ for male PwPD [21]). When comparing the control group, the current results for both females (M = 27.0 mels^2^, SD = 5.3 mels^2^) and males (M = 19.2 mels^2^, SD = 3.8 mels^2^) are comparable with the study in Dutch speakers (M = 29.5 mels^2^, SD = 7.5 mels^2^ for females; M = 20.3 mels^2^, SD = 4.1 mels^2^ for males [21]). The overall alignment in group findings by sex between the two studies, except for the female PwPD, suggests that speech symptoms were milder in the prior work for female PwPD [21]. Together with the comparability of the AAVS results to prior work in American English in kHz^2^, these collective findings suggest that AAVS is robust to connected speech across different phonetic contexts in PwPD and controls. Therefore, there is evidence that AAVS and other trajectory-based measures of vowel space are comparable across different speech stimuli and languages.

### Using token- and trajectory-based vowel space measures for speaker group detection

For the second aim, the vowel space metrics performed relatively poorly when discriminating speaker groups (*R*^2^ < 0.113). This result suggests that acoustic features of vowel space alone may be insufficient for adequate group classification, or that a wider range in dysarthria severity is required. This argument is strengthened by evidence that combining speech metrics across articulation, phonation, and prosody shows better performance than individual metrics when classifying PwPD and controls [46] and that vowel space metrics are sensitive to typical vs. dysarthric speech when using a sample across different speech disorders [18]. Thus, when considering speech acoustics as a possible marker of PD status, measures of speech that reflect the disruptions in symptoms across all three subsystems (respiratory, laryngeal, and articulatory [2]) seem to be necessary to achieve increased classification accuracy.

### Relations between vowel space measures and intelligibility

For the third aim, token-based metrics were statistically associated with perceived speech intelligibility. The tVSA and VAI models suggested a better fit compared to the base model using only group and sex as predictors of speech intelligibility (reductions in AICc of 7.5 and 8 for the tVSA and VAI models, respectively). The AAVS model yielded an AICc reduction of 3.3, however the AAVS was not a statistical predictor of speech intelligibility. This is in line with prior work comparing vowel space measures in their ability to predict speech intelligibility in speakers with a variety of motor speech disorders (PD, amyotrophic lateral sclerosis, Huntington’s disease, and cerebellar ataxia), which found that VSA was the best predictor [4]. The authors assessed token-based measures (VSA and corner dispersion) and trajectory-based measures (vowel space hull area and vowel space density) in relation to both orthographic transcription and VAS ratings of speech intelligibility. The authors found the token-based VSA model was best at predicting VAS ratings of intelligibility (*R*^2^ = 0.49) when compared to the other acoustic measures [4]. Taken together, the combined studies support that token-based measures of vowel space, specifically tVSA and VSA, are strong predictors of speech intelligibility. Although the current study was specific to PwPD, the prior study of relations between vowel space metrics and intelligibility included speakers with different motor speech disorders [4]; thus, token-based metrics of vowel space appear to be well-suited for assessing speech intelligibility changes across various speech disorders.

The relationships between individual vowel space metrics and speech intelligibility were relatively weak (*R*^2^ < 0.35). This outcome is somewhat expected, as vowel space metrics alone cannot capture the speech changes present across all speech subsystems in PwPD, and weak relationships between individual vowel space metrics and speech intelligibility were also found in prior work [4]. Vowels only capture one aspect of speech changes that reduce perceived intelligibility. Imprecise consonant articulation, which has been implicated in PwPD [2], can also influence perceived speech intelligibility. Nonetheless, this work supports that token-based measures such as VAI or tVSA, paired with metrics reflecting other speech subsystems, are well-suited for follow-up studies aiming to quantify perceptual intelligibility for PwPD.

As noted earlier, the range in speech severity within the group of speakers assessed is important to consider for acoustic and perceptual comparisons. Given that the current sample had a wide range in speech intelligibility, it remains unclear whether vowel space metrics are sensitive to milder changes in speech intelligibility at an individual-level for PwPD or other motor speech disorders. This question should be explored in future work to determine best-suited metrics for tracking changes in speech intelligibility within speakers over time.

### Limitations

This study sought to improve our understanding of vowel space metrics for studying acoustic and perceptual changes to speech in PwPD, but there were limitations of this investigation. Firstly, the current work assessed three acoustic measures of vowel space based on their demonstrated effectiveness for quantifying speech changes and perceived intelligibility in PwPD; however, there are several measures of vowel space area that could be explored in terms of their ability to detect changes across a variety of speech conditions. Thus, conclusions could only be made based on the measures included. Future work could explore larger studies that compare different motor speech disorders with a wider range in speech severity and numbers of speech metrics assessed. Additionally, there are still several barriers to clinical applicability (e.g., time burden, training requirements, automation potential, stability metrics, and minimal detectable change) that need to be addressed to assess whether these vowel metrics are also clinically meaningful.

Another limitation is that the vowel space measures and the intelligibility ratings were calculated from differential speech stimuli. Moreover, two PwPD in the study did not record the SIT sentences, so a set of five separate unique connected speech sentences extracted from other stimuli were used for these speakers. Although all stimuli involved reading connected speech, the differing phonetic content could have affected the strength of the relationship between the acoustic measures and perceptual intelligibility ratings. This methodological choice was done to prevent familiarization effects during the intelligibility rating task, since the vowel space metrics were calculated from a custom passage reading that was the same across all speakers. As a result, the current methods also extended previous findings of a relation between vowel space measures and intelligibility ratings using the same stimuli [4] to assess whether the relationship remains when calculated from differential stimuli. Despite the possible effects of differing connected speech stimuli on the relation between vowel space and intelligibility, a statistical relationship was found for token-based metrics. This result suggests that speech elicited across different sentence reading contexts are similarly reflective of speech intelligibility changes in PwPD when using token-based metrics. However, it is unclear how the current results would generalize to other types of connected speech (e.g., a picture description task or spontaneous speech) so this possibility needs further investigation.

For the AAVS calculations, it is possible that the length of the sample influenced the study findings. Namely, continuous vowel formants across two sentences may not be as representative as a longer speech utterance (e.g., a full reading passage). Prior work found that AAVS across a single sentence of the Rainbow passage was sensitive to changes in speech clarity [7,10]; however, the influence of the length of the speech sample on AAVS calculations should be further explored in future work.

The current work is also limited by the inclusion of one language group (American English), so it was not possible to explore possible linguistic influences on the sensitivity of vowel space area metrics to speech changes in PwPD. Given the conflicting findings across languages (reviewed above) for the sensitivity of the vowel metrics in PwPD, and toward improving global speech assessment for PwPD, additional work is needed to determine sensitive acoustic metrics for PwPD in more languages.

### Conclusion

When assessing vowel space metrics that are token-based (triangular vowel space area, tVSA, and the vowel articulation index, VAI), and trajectory-based (articulatory-acoustic vowel space, AAVS) from the same stimuli, the current work found that: (1) all metrics yielded group differences between PwPD and controls, especially for female speakers; (2) all three acoustic measures performed poorly at distinguishing speaker groups; and (3) token-based metrics were associated with perceived speech intelligibility. Future work is needed to assess whether these findings are generalizable to other motor speech disorders as well as across different speech stimuli and languages.

## Supporting information

S1 File Analyzed dataset. Dataset of formant values used for the vowel space measures.
(XLSX)

## References

[1] Coates C, Bakheit AM. The prevalence of verbal communication disability in patients with Parkinson’s disease. Disabil Rehabil. 1997;19(3):104–7. doi: 10.3109/09638289709166834 9134353

[2] Broadfoot C, Abur D, Hoffmeister J, Stepp C, Ciucci M. Based updates in swallowing and communication dysfunction in parkinson disease: implications for evaluation and management. Perspect ASHA Spec Interest Groups. 2019;4(5):825–41. 32104723 10.1044/2019_pers-sig3-2019-0001 PMC7043100

[3] Ferguson SH, Kewley-Port D. Talker differences in clear and conversational speech: Acoustic characteristics of vowels. 2007. 10.1044/1092-4388(2007/087) 17905909

[4] Thompson A, Hirsch ME, Lansford KL, Kim Y. Vowel acoustics as predictors of speech intelligibility in dysarthria. J Speech Lang Hear Res. 2023;66(8S):3100–14. doi: 10.1044/2022_JSLHR-22-00287 36795536 PMC10569402

[5] Skodda S, Grönheit W, Schlegel U. Impairment of vowel articulation as a possible marker of disease progression in Parkinson’s disease. PLoS One. 2012;7(2):e32132. doi: 10.1371/journal.pone.0032132 22389682 PMC3289640

[6] Tjaden K, Lam J, Wilding G. Vowel acoustics in Parkinson’s disease and multiple sclerosis: comparison of clear, loud, and slow speaking conditions. J Speech Lang Hear Res. 2013;56(5):1485–502. doi: 10.1044/1092-4388(2013/12-0259 23838989 PMC5572218

[7] Whitfield JA, Goberman AM. Articulatory-acoustic vowel space: application to clear speech in individuals with Parkinson’s disease. J Commun Disord. 2014;51:19–28. doi: 10.1016/j.jcomdis.2014.06.005 25074511

[8] Sapir S, Ramig LO, Spielman JL, Fox C. Formant centralization ratio: a proposal for a new acoustic measure of dysarthric speech. J Speech Lang Hear Res. 2010;53(1):114–25. doi: 10.1044/1092-4388(2009/08-0184 19948755 PMC2821466

[9] Sapir S, Fox C, Spielman J, Ramig L. Acoustic metrics of vowel articulation in Parkinson’s disease: vowel space area (VSA) vs. vowel articulation index (VAI). In: Models and analysis of vocal emissions for biomedical applications: 7th international workshop: August 25–27, 2011, Firenze, Italy: Firenze University Press; 2011.

[10] Whitfield JA, Goberman AM. Articulatory-acoustic vowel space: associations between acoustic and perceptual measures of clear speech. Int J Speech Lang Pathol. 2017;19(2):184–94. doi: 10.1080/17549507.2016.1193897 27328115

[11] Weismer G, Jeng JY, Laures JS, Kent RD, Kent JF. Acoustic and intelligibility characteristics of sentence production in neurogenic speech disorders. Folia Phoniatr Logop. 2001;53(1):1–18. doi: 10.1159/000052649 11125256

[12] McRae PA, Tjaden K, Schoonings B. Acoustic and perceptual consequences of articulatory rate change in Parkinson disease. J Speech Lang Hear Res. 2002;45(1):35–50. doi: 10.1044/1092-4388(2002/003 14748637

[13] DeSilva GS, Upadhyay P, Manxhari M, Gopal D, Smith KM. Variability in vowel space in Parkinson’s Disease: associations with cognitive and motor impairment. J Speech Lang Hear Res. 2024;67(10):3566–78. doi: 10.1044/2024_JSLHR-24-00008 39259881 PMC11482582

[14] Whitfield JA, Mehta DD. Examination of clear speech in Parkinson disease using measures of working vowel space. J Speech Lang Hear Res. 2019;62(7):2082–98. doi: 10.1044/2019_JSLHR-S-MSC18-18-0189 31306606

[15] Skodda S, Visser W, Schlegel U. Vowel articulation in Parkinson’s disease. J Voice. 2011;25(4):467–72. doi: 10.1016/j.jvoice.2010.01.009 20434876

[16] Roland V, Huet K, Harmegnies B, Piccaluga M, Verhaegen C, Delvaux V. Vowel production: a potential speech biomarker for early detection of dysarthria in Parkinson’s disease. Front Psychol. 2023;14:1129830. doi: 10.3389/fpsyg.2023.1129830 37701868 PMC10493417

[17] Rusz J, Cmejla R, Tykalova T, Ruzickova H, Klempir J, Majerova V, et al. Imprecise vowel articulation as a potential early marker of Parkinson’s disease: effect of speaking task. J Acoust Soc Am. 2013;134(3):2171–81. doi: 10.1121/1.4816541 23967947

[18] Lansford KL, Liss JM. Vowel acoustics in dysarthria: speech disorder diagnosis and classification. J Speech Lang Hear Res. 2014;57(1):57–67. doi: 10.1044/1092-4388(2013/12-0262 24687467 PMC4096018

[19] Rusz J, Cmejla R, Ruzickova H, Ruzicka E. Quantitative acoustic measurements for characterization of speech and voice disorders in early untreated Parkinson’s disease. J Acoust Soc Am. 2011;129(1):350–67. doi: 10.1121/1.3514381 21303016

[20] Story BH, Bunton K. Vowel space density as an indicator of speech performance. J Acoust Soc Am. 2017;141(5):EL458. doi: 10.1121/1.4983342 28599542 PMC5724721

[21] Hoekzema N, Rebernik T, Tienkamp TB, Chaboksavar S, Ciot V, Gleichman A. Assessing differences in articulatory-acoustic vowel space in Parkinson’s disease by sex and phenotype. Seminar on Speech Production (ISSP); 2024.

[22] Tienkamp T, Rebernik T, Jacobi J, Wieling M, Abur D. The impact of electromagnetic articulography sensors on the articulatory-acoustic vowel space in speakers with and without Parkinson’s disease. In: 13th International Seminar of Speech Production; 2024.

[23] Houle N, Feaster T, Mira A, Meeks K, Stepp CE. Sex Differences in the speech of persons with and without Parkinson’s disease. Am J Speech Lang Pathol. 2024;33(1):96–116. 37889201 10.1044/2023_AJSLP-22-00350 PMC11000784

[24] Bang Y-I, Min K, Sohn YH, Cho S-R. Acoustic characteristics of vowel sounds in patients with Parkinson disease. NeuroRehabilitation. 2013;32(3):649–54. doi: 10.3233/NRE-130887 23648619

[25] Arias-Vergara T, Vásquez-Correa JC, Orozco-Arroyave JR. Parkinson’s disease and aging: analysis of their effect in phonation and articulation of speech. Cogn Comput. 2017;9(6):731–48. doi: 10.1007/s12559-017-9497-x

[26] Abur D, Subaciute A, Daliri A, Lester-Smith RA, Lupiani AA, Cilento D, et al. Feedback and feedforward auditory-motor processes for voice and articulation in Parkinson’s disease. J Speech Lang Hear Res. 2021;64(12):4682–94. doi: 10.1044/2021_JSLHR-21-00153 34731577 PMC9150666

[27] Schow RL. Considerations in selecting and validating an adult/elderly hearing screening protocol. Ear Hear. 1991;12(5):337–48. doi: 10.1097/00003446-199110000-00006 1783237

[28] Dahl KL, Balz MA, Cádiz MD, Stepp CE. How to efficiently measure the intelligibility of people with Parkinson’s disease. Am J Speech Lang Pathol. 2025;34(1):70–84. doi: 10.1044/2024_AJSLP-24-00080 39475678 PMC11745308

[29] American Speech-Language-Hearing Association. Guidelines for manual pure-tone threshold audiometry. Rockville, MD: American Speech-Language-Hearing Association; 2005.

[30] Beukelman DR, Yorkston KM, Hakel M, Dorsey M. Speech intelligibility test. Lincoln: Madonna Rehabilitation Hospital; 2007.

[31] Bunton K. Fundamental frequency as a perceptual cue for vowel identification in speakers with Parkinson’s disease. Folia Phoniatr Logop. 2006;58(5):323–39. doi: 10.1159/000094567 16966834

[32] Tjaden K, Sussman JE, Wilding GE. Impact of clear, loud, and slow speech on scaled intelligibility and speech severity in Parkinson’s disease and multiple sclerosis. J Speech Lang Hear Res. 2014;57(3):779–92. doi: 10.1044/2014_JSLHR-S-12-0372 24687042 PMC5564324

[33] Abur D, Enos NM, Stepp CE. Visual analog scale ratings and orthographic transcription measures of sentence intelligibility in Parkinson’s disease with variable listener exposure. Am J Speech Lang Pathol. 2019;28(3):1222–32. doi: 10.1044/2019_AJSLP-18-0275 31296027 PMC6802923

[34] Olsen WO. Average speech levels and spectra in various speaking/listening conditions: a summary of the Pearson, Bennett, & Fidell (1977) report. Am J Audiol. 1998;7(2):21–5. doi: 10.1044/1059-0889(1998/012 26649514

[35] Kent RD, Weismer G, Kent JF, Rosenbek JC. Toward phonetic intelligibility testing in dysarthria. J Speech Hear Disord. 1989;54(4):482–99. doi: 10.1044/jshd.5404.482 2811329

[36] Boersma P, Weenink D. Praat: doing phonetics by computer; 5.3-6.0 ed. 2016.

[37] MathWorks. MATLAB 2016. b ed. Natick, Massachusetts, United States: MathWorks. 2016.

[38] Barsties B, Maryn Y. External validation of the acoustic voice quality index version 03.01 with extended representativity. Ann Otol Rhinol Laryngol. 2016;125(7):571–83. doi: 10.1177/0003489416636131 26951063

[39] Tienkamp TB, Rebernik T, Halpern BM, van Son RJJH, Wieling M, Witjes MJH, et al. Associations between acoustic, kinematic, self-reported, and perceptual measures of speech in individuals surgically treated for oral cancer. J Speech Lang Hear Res. 2025;68(7):3069–89. doi: 10.1044/2025_JSLHR-24-00464 40570236

[40] Sussman JE, Tjaden K. Perceptual measures of speech from individuals with Parkinson’s disease and multiple sclerosis: intelligibility and beyond. J Speech Lang Hear Res. 2012;55(4):1208–19. doi: 10.1044/1092-4388(2011/11-0048 22232396 PMC5564315

[41] Stipancic KL, Tjaden K, Wilding G. Comparison of intelligibility measures for adults with Parkinson’s disease, adults with multiple sclerosis, and healthy controls. J Speech Lang Hear Res. 2016;59(2):230–8. doi: 10.1044/2015_JSLHR-S-15-0271 26556727 PMC4972008

[42] Convey RB, Ihalainen T, Liu Y, Räsänen O, Ylinen S, Penttilä N. A comparative study of automatic vowel articulation index and auditory-perceptual assessments of speech intelligibility in Parkinson’s disease. Int J Speech Lang Pathol. 2024;26(5):663–73. doi: 10.1080/17549507.2023.2251725 37800979

[43] Castillo-Triana N, Camargo-Mendoza M. Vowel articulation and intelligibility of speech in Spanish speakers with Parkinson’s disease treated with deep brain stimulation of the subthalamic nucleus. Rev Neurol. 2024;79(5):121–7. doi: 10.33588/rn.7905.2024108 39207126 PMC11469106

[44] Whitfield JA, Dromey C, Palmer P. Examining acoustic and kinematic measures of articulatory working space: effects of speech intensity. J Speech Lang Hear Res. 2018;61(5):1104–17. doi: 10.1044/2018_JSLHR-S-17-0388 29710247

[45] Novotny M, Rusz J, Cmejla R, Ruzicka E. Automatic evaluation of articulatory disorders in Parkinson’s disease. IEEE/ACM Trans Audio Speech Lang Process. 2014;22(9):1366–78. doi: 10.1109/taslp.2014.2329734

[46] Rios-Urrego CD, Rusz J, Orozco-Arroyave JR. Automatic speech-based assessment to discriminate Parkinson’s disease from essential tremor with a cross-language approach. NPJ Digit Med. 2024;7(1):37. doi: 10.1038/s41746-024-01027-6 38368458 PMC10874421

